# Aging and cancer: Clinical role of tumor markers in the geriatric population (Review)

**DOI:** 10.3892/mi.2024.145

**Published:** 2024-03-06

**Authors:** Sivapatham Sundaresan, Palanirasu Rajapriya, Selvaraj Kaveri Lavanya

**Affiliations:** 1 Department of Medical Research, SRM Medical College Hospital and Research Centre, SRM Institute of Science and Technology, SRM Nagar, Kattankulathur, Tamil Nadu 603203, India; 2 Department of Liver Sciences, Rela Institute of Medical Sciences, Chennai, Tamil Nadu 600044, India

**Keywords:** tumor markers, aging, geriatrics, cancer, genomic instability, age and sex specificity

## Abstract

Aging, with the progressive deterioration and functional decline of several organ systems, is highly heterogeneous for both between and within individuals. Tumor markers are widely used in clinical practice as a screening test for individuals >50 years of age. More specifically, caring for elderly patients is a public health concern, given the incidence of cancer and its related mortality and morbidity. A multidisciplinary diagnostic procedure known as a geriatric assessment is capable of identifying functional, psychological and physiological issues that are missed by standard evaluation. The present review focuses on cancers affecting the geriatric population, highlights current opportunities and challenges, and highlights the unmet need for clinically relevant tumor markers in elderly patients with cancer. A comprehensive geriatric examination, including a biological assessment, still requires conveniently available tumor markers and their levels in older populations in order to forecast deterioration or loss of functional balance. These tumor indicators ought to make it possible to track patients using other outcomes, such overall survival and functional impairment. Despite the notable progress made in the understanding of human biology, the mechanisms and networks underlying aging remain largely unknown. In addition, as elderly patients are a highly heterogeneous population, age-related changes cannot be distinguished solely by chronological age. Strong clinical studies, well-established protocols and meta-analyses may contribute to the better utilization of tumor biomarkers in the elderly population. Hence, the present review addresses the effects of aging on tumor markers and the usefulness of tumor marker values for the geriatric population.

## 1. Introduction

Cancer is the second leading cause of mortality worldwide, with an estimated 19.3 million new cancer cases (1). Due to the exponentially higher incidence of cancer in later life, 60% of newly diagnosed malignancies and 70% of cancer-related deaths occur in patients aged >65 years (2-3). By 2040, population growth and aging alone are expected to increase the global burden to 27.5 million new cancer cases and 16.3 million cancer-related deaths (4). However, the prevalence of risk-increasing factors, such as smoking, an unhealthy diet and a lack of physical activity could significantly increase the future cancer burden (5). Cancer is primarily a disease of the elderly, with >80% of cancer cases arising in humans >50 years. As this amount of the population is likely to increase gradually over several decades, an urgent need to address this impending health and medical crisis by developing policies to decrease cancer rates in older adults. Evolving suitable plans will require recognizing individuals at risk of developing cancer and providing them with specific risk-reduction procedures that go above current endorsements for prevention and screening. Monogenic and polygenic effects play a crucial role in cancer susceptibility and pathogenesis; however, cancer in the elderly is less expected to be related to single-germline variant genes (4). The degree to which polygenic variants precisely contribute to the risk of cancer development is the topic of the present review.

The fundamental processes that mediate susceptibility to cancer, which are common among all cancers are also likely to mediate vulnerability to other non-cancerous diseases and conditions. These developments are most likely related to aging and longevity, due to the appearance of a number of common age-related chronic diseases. By bringing together genetic information about multiple cancers, diseases, aging and life expectancy, it is possible to develop better predictive models through risk stratification, and to also learn more about the influences of aging and the genetic causes of cancer. Tumors arise from the oncogenic transformation changes of only single cells. Some tumors gain the ability to leave their site of origin and invade other bodily organs (6). As with all diagnostic tests, tumor markers help doctors determine whether it is a new cancer, recurrence, cancer progression or mortality, and/or specific treatments to reduce risk. They are a very useful surrogate indicator (7). The value of tumor markers is to enable the more efficient application of therapy, thus allowing treatment to be used in patients most likely to benefit, while reducing toxicity in those who do not benefit and reducing exposure (8). Despite the fact that data on the histological scoring of breast tumor subtypes and prostate cancer have enabled the investigation of long-term associations with risk factors, a dilution effect could not be completely ruled out. However, this may have resulted in a non-differential measurement error (both in cases and non-cases), which most likely would have understated the strength of the associations that were present.

## 2. Biological aging and cancer tumor markers

An area of particular interest in biological aging mechanisms, which may constitute a shared set of pathways that increase the likelihood of developing a variety of diseases normally associated with aging, including cardiovascular disease, is numerous common forms of cancer and degenerative diseases (‘geriatric science hypothesis’) (9). Biomarkers of aging can be used to forecast the overall risk of chronic disease and offer new insight into the causes of diseases and their underlying biological pathways (10). Furthermore, increasing evidence suggests that biological aging mechanisms and processes can be specifically targeted and moderated by preventive and therapeutic interventions, including alterations in dietary intake and energy restriction, and increased physical exercise or drug therapy (11). The biological aging process is related to a series of biological variations. At the cellular level, this includes amplified genomic instability, epigenetic alterations, mitochondrial dysfunction, higher oxidative stress, protein imbalance, impaired nutrient perception and senescence cell chemistry (12). At a more systemic level, aging is categorized by a loss and physiological changes in respiratory, cardiovascular, neurological, metabolic, musculoskeletal, liver and renal functions (13), which may be mirrored in the various circulating blood biomarkers (14).

One of the main risk factors of cancer is aging. In a World Health Organization report, the incidence of cancer was examined across various age groups. The prevalence of other cancers rises based on diabetes [breast (post-menopausal) and pancreatic cancers], infections (hepatocellular carcinoma and anal cancer), weight and metabolic syndrome [esophageal, pancreatic, thyroid, gallbladder, colon and rectal, breast (post-menopausal), endometrial and kidney cancers] and sharply with age; unlike leukemia, which mostly affects younger individuals, a strong link has been found between cancer and aging, suggesting that cancer is an aspect of aging (15). Telomere shortening, an accumulation of genetic mutations, oxidative stress and the breakdown of cells and organs, are only a few of the numerous elements that contribute to aging, a complex and universal physiological process. The capacity of the body to maintain a stable state steadily declines with age, and the chance of developing diseases such as cancer, cardiovascular issues and neurological conditions also increases (16). Nevertheless, due to the limited genetic backdrop, the human aging processes is not completely understood. Tumor markers play a crucial role in patient monitoring. A clinical issue that increases the reliability and utility of tumor markers is the accuracy of the marker results for the desired submission. Marker results classify patients into two or more populations with markedly dissimilar results, and patients and their caregivers are treated as distinct groups (17). The incidence of tumors in the aging population is high, and hence older individuals are keen to participate in health programs (18). However, although cancer and senescent cells are also basically opposed, the underlying mechanism of both cancer and senescence is the time-dependent accumulation of cellular damage (19). Irrespective of the mechanisms or networks underlying the aging process, or the criteria used to define patients as ‘old’, aging itself is considered to be the most significant risk factor for the growth of unfavorable cancers. When interpreting laboratory data, it is crucial to take into account the effects of age in order to correctly evaluate the results; however, whether or not aging alone is accountable for changes in certain biological pointers and whether the aberrations are due to pathological causes or degenerative diseases with a high prevalence in the elderly population are questionable (20).

## 3. Tumor markers and the elderly

To demonstrate the potential association between cancer and aging, a large-scale genome-wide study of DNA methylation profiles in aging and cancer was previously completed (21). Applying an age and cancer-related weighting network produces biologically meaningful results, representing that the association between age-related variations in DNA methylation and carcinogenesis is not accidental (22). Age is a risk factor for cancer; however, the association between age and cancer is unclear. Increased or decreased methylation is detected up to the age of 70, although these phenomena plateau with aging in healthy populations. Molecular markers are DNA fragments that signify genetic signatures for detecting changes in gene sequence, expression levels and protein structure or function (23). The extensive cancer research offered by genomics helps to characterize tumors at the molecular level. Similar advancements in molecular technology have elevated cancer biology. For instance, the creation of medications that target pertinent molecules has been influenced by this information. A platform for developing cancer molecular markers requires a solid understanding of the genes involved in cancer development, according to recent findings from a research project by the International Cancer Genome Consortium (ICGC) and The Cancer Genome Atlas (TCGA) (24). The process of studying these oncogenes requires both molecular therapeutics and biomarkers, leading to the growth of drugs against specific cancers (25). The number of molecular markers has multiplied over the past decade due to the better understanding of the molecular biology of cancer, as well as the molecular basis of tumor progression and therapeutic response.

There are basically three types of biomarkers (prognostic, preventive and diagnostic markers) available. The tumor biomarkers in cancer are presented in Table I (26-37). While all subtypes occur in all age categories, an older age is associated with a slightly lower incidence of high-grade tumors, fewer triple-negative breast cancer and HER2^+^ subtypes, and more luminal tumors than a younger age (38). Age-related differences in the landscape of tumor mutations include the fact that older patients with breast cancer have a lower incidence of TP53 mutations than younger patients (28). There have been reports of age-dependent alterations in peritumoral and systemic immunity; nevertheless, additional studies in various subtypes of breast cancer are necessary. With an advancing age, distinct histological (more common squamous cell carcinoma) and molecular (increased tumor mutational burden and distinct EGFR mutation subtypes) changes appear to manifest that may affect the management of lung cancer. Age groups do not appear to exhibit differences as regards the expression of programmed cell death protein 1 (PD-1) and PD-L1; however, further studies are required to fully characterize the tumor microenvironment and identify any differences between older and younger patients (39). A higher Gleason score, a higher D'Amico risk classification, more often occurring in the luminal B subtype, more common intraductal carcinoma of prostate architecture, a higher p53 positivity, and more tumors with a high-risk Decipher score all indicate that prostate cancer in older males generally appears to behave more aggressively (35,40). The interpretation of serum prostate-specific antigen is hampered by aging, which is why age-based standards and alternative biomarkers are used. When compared to younger individuals, elderly patients with colorectal cancer exhibit notable biochemical changes. Right-sided tumors are more common among older adults, and serrated polyps, as opposed to the traditional adenoma-carcinoma pathway, are more frequently linked to the development of cancer. In light of the increasing use of targeted therapies and immunotherapy, there is a larger frequency of BRAF mutations, microsatellite instability phenotype and CpG island methylator phenotype-high tumors at the molecular level (41). These findings may have significant therapeutic consequences. The predictive value of an immunoscore appears to be age-independent (42).

## 4. Prognostic, predictive and diagnostic markers

Prognostic markers assess the complete outcomes of patients following typical therapy and predict disease progression or the response to therapeutic intervention in subjects with similar characteristics (43). Predictive markers quantity the usefulness of a particular clinical intervention, or different outcomes of two or more interventions, and specify sensitivity or resistance to a particular therapy. Diagnostic markers, on the other hand, recognize whether a patient is suffering from a particular disease by measuring susceptibility or resistance to a particular treatment. Biomarkers help assess preventive and therapeutic measures and detect early stages of malignant transformation of the oral mucosa. Biomarkers reveal genetic and molecular alterations associated with early, intermediate and late endpoints of the oral carcinogenesis process. Circulating biomarkers, such as circulating tumor DNA, exosomes and circulating microRNAs (miRNAs/miRs) can be used for early prognosis, diagnosis and treatment. Circulating miRNAs, such as miR-138, miRa-99a, miR-21, miR-181a, miR-222 and miR-7, have enhanced the diagnostic ability for head and oral cancer (44). Pharmaceutical research and development are focused on oncology. The discovery of therapeutic breakthroughs in oncology accounts for 29% of the total R&D spending. Given that cancer is a highly heterogeneous disease, not only in terms of histology and clinical outcomes, but also at the molecular level, oncology is one of the first fields to use targeted therapy (45). Tumor barkers and their specific genetic alterations are summarized in Table II (46-55).

Increased markers in the elderly do not reflect the presence of tumors, but may have some association with the carcinogenic process. The starting point may be the increase in multiple hyperplasia that occurs with aging and rarely leads to clinical neoplasms (56). Hyperplasia in the epithelial cells of the gastrointestinal tract has been widely studied and has been revealed to be closely related with stomach cancer, colonic metaplasia and age, with a noticeable increase in premalignant abrasions with age (57). Another possibility is that the markers merely represent age-related, non-specific organ degeneration (57). To measure risk aspects and predict efficacy/toxicity, treatment tolerability and overall risk/benefit ratio in elderly patients are critical parameters to consider in the decision-making of patients with cancer. Additionally, clinically relevant indicators are required for the delivery of xenobiotics. There currently limited data available due to the dearth of age-specific effectiveness and safety data in clinical trials (58). Elevated marker values are not due to the increased prevalence of chronic disease and not due to the presence or preclinical stage of occult malignancies, in most cases. The study by Lopez *et al* (60) compared the decreased sensitivity of the markers in subjects without clinical signs of cancer; it was noted that the increase was not associated with the presence of antigen-associated tumors. Circulating cytokines may be related to the increased mortality in aging patients with cancer. Potential biomarkers require extensive investigation before being validated for clinical use (59). Comprehensive geriatric assessment as an interdisciplinary framework for assessing the impact of age-related physiological factors, as divergent to chronological age, that may affect health and disease in older adults was developed (60). Those identified at risk by the screening test need to be further evaluated with a full CGA, and the results used may help inform specific healthcare strategies to improve cancer outcomes in older adults (61). There is also an understanding of the metabolic and molecular changes associated with cancer. Elderly patients continue to be underrepresented in clinical trials, making evidence-based decision-making for older patients difficult. In particular, race, sex and accounting for age-related inequalities remain subjects of debate concerning participation in cancer clinical trials. There is an urgent need to better understand the clinical, molecular and physiological implications of cancer in the elderly and the factors that regulate treatment response, toxicity, and tolerability in older patients with cancer (62).

## 5. Genomic instability in aging cells

**Figure 1:**
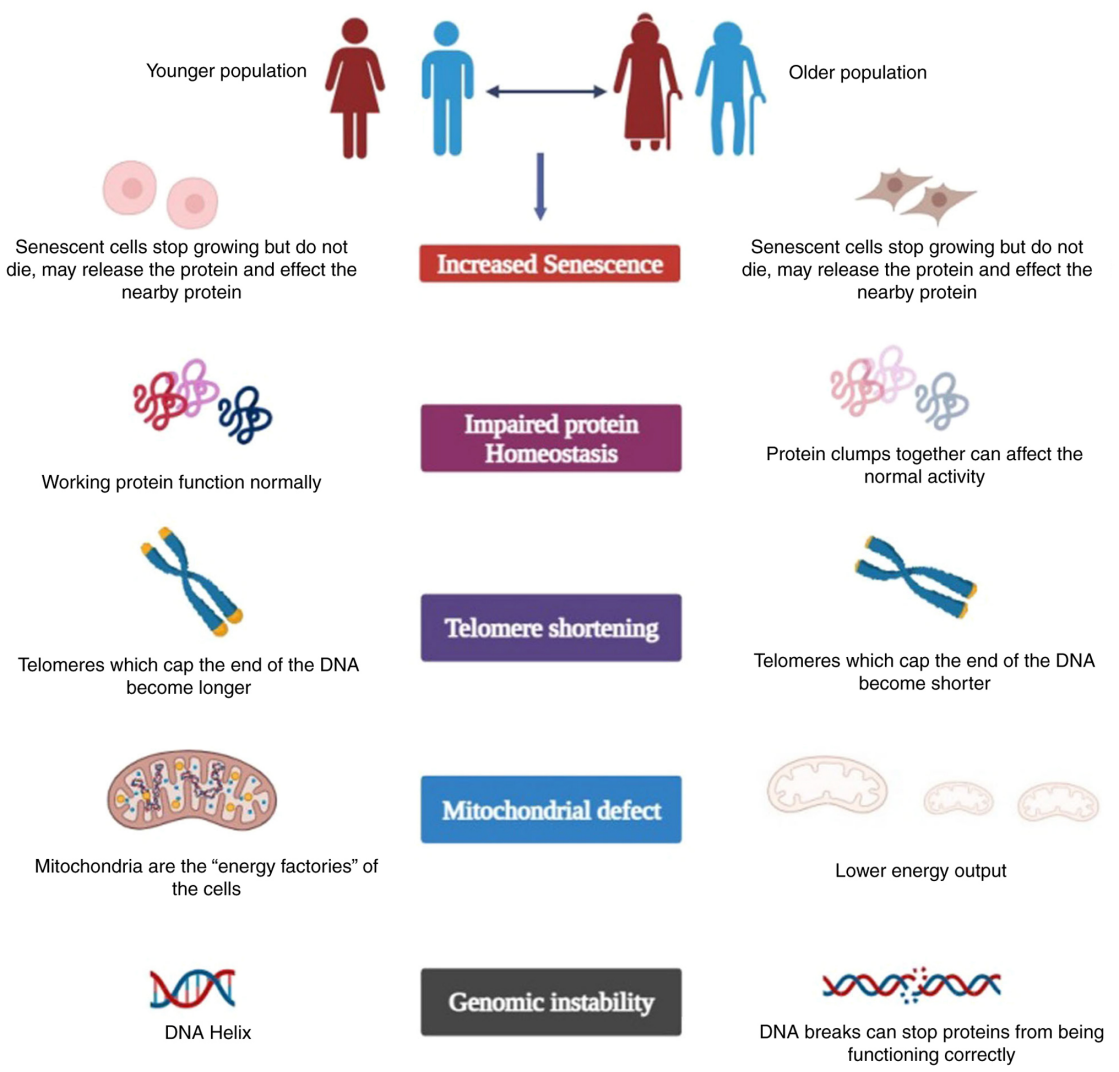
Schematic representation of the mechanisms involved in aging and cancer.

Mutations contribute to tumorigenesis, almost all human cancers exhibit genomic instability, and mutations occur at a greater pace than in healthy cells. Preserving genomic stability appears to be an essential function for preventing cancer progression and aging processes. Alterations and instability in the genome can contribute to aging via a variety of mechanisms, from minor point mutations to significant translocations and deletions. Alterations in regulatory sequences can lead to a progressive decline in organ function as a result of variations in the proteome and homeostasis (63). Indeed, there is a significant age-related disparity in the expression of genes among cells in the same tissue. The gene heterogeneity is considered to cause the stochastic deregulation of gene expression amongst adjacent cells, which ultimately accelerates aging (64). It is conceivable that gradual alterations may occur over time, triggered by somatic mutations caused by genomic instability. These individual variations in mutations may have an effect on shared biological pathways or gene regulatory networks, which eventually result in age-related functional decline and universally prevalent disease patterns. Diseases can be caused by errors in genes that maintain genome stability and repair DNA. Additionally, as individuals age, somatic mutations spread across tissues, disrupting important transcriptional programs, as well as other vital biological functions, leading to a loss of fitness and organismal deterioration. Overall, it appears that the argument that DNA damage and genomic instability are key causes of aging and aging-related disorders, such as cancer, is becoming more and more compelling (65). Cancer, on the other hand, is the consequence of advantageous mutations that provide the neoplastic cell with an advantage in terms of growth and metastasis. In order to delay disease development and premature aging, genomic stability must be maintained. Human syndromes of early aging are caused by mutations in DNA repair enzymes (66), and genomic instability is a key characteristic of cancer (67). Numerous studies have linked DNA shape and organization to genomic instability and aging, highlighting the significance of how DNA is packed within our cells (68). Importantly, a number of signaling pathways and molecular functions, such as insulin signaling, mTOR signaling, cellular senescence (69), telomere shortening and sirtuin activity (70), have also been connected to organismal aging. However, it also signifies the stochastic hazard of accumulating changes that reassure uncontrolled cell division and expansion in cancer. Finally, therapeutic interventions that target both aging as a process and carcinogenesis may be probable with a deeper understanding of genomic instability (71,72). A schematic representation of aging and cancer is presented in Fig. 1.

## 6. Age-stratified and sex-specific reference intervals

Specific characteristics of the research participants, including their age, sex, region and way of life should be taken into consideration in order to establish a reliable and well-designed reference interval. These factors may have an effect on the levels of biomarkers in individuals who will be the focus of the investigation. When determining a reference interval that performs well, another key consideration is the volume of the sample that will be included in the study. Although the Clinical and Laboratory Standards Institute 24 (CLSI24) needs at least 120 samples to meet the sample volume requirement for founding a reference interval considering the cost of conducting, a larger volume sample, will, if the budget permits, provide a better Poisson distribution and represent a ‘near-true’ population value. A standardized assessment of tumor markers on a large population with age-, sex- and geographic location-specific well-representation of healthy subjects is compulsory to carry out a reference interval study for a tumor biomarker. It is difficult to simultaneously establish a reference interval using a multi-marker lung cancer biomarker panel in numerous hospitals (73). However, the majority of the previous studies included older women, a population that is more likely to be associated with suspicion of cancer. Patients with dermoid cysts have different clinical features depending on their age, according to an age-focused study. The likelihood of developing cancer increased after the age of 40, and tumor size was higher in younger patients than in older women (74-76). No association between was found between elevated cancer antigen 125 used for the diagnosis of ovarian, endometrial, peritoneal and fallopian tube cancers and carbohydrate antigen 19-9 for colon, stomach and bile duct cancer levels in adolescents and young adults. On the other hand, in the older age group, an increase in the levels of carbohydrate antigen 19-9 was associated with larger tumors (77). In a similar manner, the majority of studies that have already been published and found a link between carbohydrate antigen 19-9 levels and tumor size displayed a higher median age (78,79).

The dynamics of telomeres plays a crucial role in aging, age-related diseases and cancer. Stress, depression, smoking and exercise are instances of non-genetic factors that affect telomere maintenance (80).

It has been well-established that hereditary telomere disorders are caused by single-gene inactivating mutations in telomere maintenance components. Typically, these modifications cause *in vivo* telomeres to shorten. The classic phenotypes of accelerated aging include diabetes, cardiovascular disease, graying of hair, an altered skin colour, loss of immunological function and susceptibility to specific malignancies. Males have shorter telomeres than females at any age, according to previous research, and >50% of telomere length is inherited (14,81). In addition, telomere length is heritable. It has been found that age-related telomere attrition is accelerated in patients with cancer who are diagnosed later in life, although the attrition reduces 3 to 4 years prior to the diagnosis, resulting in longer telomeres (82). This could indicate that telomere shortening plays a role in early carcinogenesis before cancer hijacks and initiates telomerase activation and other methods of telomere elongation (83). This may also affect blood leukocytes, which are critical for the initiation and development of cancer. If this is validated in further research, it may be used as an early biomarker for cancer identification. Numerous age-related disorders include protein aggregation build-up and associated harmful consequences, and proteostasis is affected. Cancer stops this process by increasing the activity of the proteasome, lysosome and chaperone systems. All paths are inhibited in oncological therapy, and new technologies and drugs are constantly being developed (83). Cancer and aging are basically different, as malignant cells avoid senescence by producing additional mutations, such as the deletion of tumor suppressors (p16INK4a or p53), an example of antagonistic pleiotropy, whereas accumulating DNA damage typically will cause an increase in cell cycle inhibitors leading to senescence or apoptosis (84). In conclusion, cancer and aging are connected in time and mechanism, and a number of the same drugs and strategies can be used to target both. On the other hand, antagonistic pleiotropy can work, and inhibiting one can cause the other to be activated.

## 7. Conclusion and future perspectives

In addition, despite the fact that previous studies have established strong associations between these markers and the risk of mortality and chronological age, they cannot be interpreted as indicators of biological aging. Additionally, there has been no cross-validation of the multi-marker combination. Despite the fact that the markers are linked to aging, it is difficult to draw a clear conclusion about how closely these associations relate to any one aspect of biological aging. This is due to the fact that these markers are not only linked to aging, but may also reflect other causal pathways, such as cellular stress, cardiovascular health, glucose intolerance, inflammation, or renal dysfunction. While several of the analyses had a reduced statistical power due to the limited number of cases needed to identify links in stratified and tumor subtypes/grades models, the aim of the present review was to summarize the data obtained to date. In order to more efficiently determine when and how to treat older patients with cancer, it is important to discriminate between chronological age, physiological age, and associated geriatric and comorbidities. Of note, multidisciplinary research including geriatricians, oncologists and policymakers, with more data on the elderly population, is warranted in order to improve clinical decision-making. However, it appears reasonable to conclude that the relation of tumor markers with tumor size has limited diagnostic value among the elderly. Consolidating the research results of clinical data from systematic reviews and meta-analyses along with a specific focus on the values of tumor markers in geriatric patients with cancer in further studies with larger study groups may provide answers to the currently pending questions.

## Figures and Tables

**Table I:** Cancer and tumor markers.

Tumor markers	Type of cancer	Age-related differences in molecular markers	(Refs.)
Luminal a	Breast	50% ≥40	(26)
Luminal b	Breast	38% ≥70	(27)
HER2	Breast	17% ≥65	(28)
TP53	Breast	42% ≥40-65	(29)
GATA3	Breast	22% ≥40	(26)
EGFR	Lung	21% ≥60	(30)
PDL1	Lung	55% ≥65	(31)
KRAS	Lung	47% ≥50	(32)
Gleason score	Prostate	47% ≥50	(33)
Serum PSA	Prostate	85% ≥60	(34)
p53	Prostate	24% ≥70	(35)
MSI	Colon	19.5% ≥75	(36)
BRAF	Colon	20% ≥60	(37)

**Table II:** Tumor barkers and their specific genetic alterations.

Tumor type	Targeted therapeutics	FDA approved specific Genetic Alterations	(Refs.)
Non-small cell lung cancer	Gefitinib, crizotinib and erlotinib	• EGFR exon 19 deletions, L858R	(46,47)
		• EGFR exon 20 insertions	
		• EGFR nonresistant mutations other than exon 19 deletions and L858R	
Breast cancer	Lapatinib	• ERBB2 amplification	(48)
Metastatic melanoma	Vemurafenib, Dabrafenib	• BRAF V600E	(49)
Colorectal cancer	Panitumumab, cetuximab	• KRAS and/or NRAS exon 2, 3, and 4 mutations	(50)
Fallopian tube, ovarian, primary peritoneal carcinoma	Bevacizumab, olaparib	• Deleterious germline or somatic mutations in BRCA1 and/or BRCA2	(51)
Esophagogastric cancer	Trastuzumab, cisplatin, capecitabine or fluorouracil	• ERBB2 amplification	(52)
Endometrial cancer	Dostarlimab	• dMMR and/or MSI-H	(53)
Ovarian cancer	Niraparib	• GIS-positive or HRD-positive	(51)
Bladder cancer	Erdafitinib	• FGFR2 fusions	(54)
Cholangiocarcinoma	Pemigatinib, infigratinib	• FGFR2 fusions	(55)

## Data Availability

Not applicable.
